# Insights into microbial dysbiosis and Cutibacterium acnes CAMP factor interactions in acne vulgaris

**DOI:** 10.1099/mgen.0.001449

**Published:** 2025-07-16

**Authors:** Qi Chen, Congyu Liu, Juan Tao, Weihong Zeng, Zhongliang Zhu, Chengbing Yao, Yuhua Shang, Jun Tang, Tengchuan Jin

**Affiliations:** 1 Department of Dermatology, The First Affiliated Hospital of USTC, Division of Life Sciences and Medicine, University of Science and Technology of China, Hefei, Anhui 230001, PR China; 2 Laboratory of Structural Immunology, Key Laboratory of Immune Response and Immunotherapy, Division of Life Sciences and Medicine, University of Science and Technology of China, Hefei, Anhui, 230027, PR China; 3 School of Biomedical Pharmaceutical Sciences, Hefei Institute of Technology, Hefei, Anhui, 238076, PR China; 4 School of Life Sciences, Division of Life Sciences and Medicine, University of Science and Technology of China, Hefei, Anhui, 230027, PR China; 5 Anhui Genebio Biotechnology Company, Hefei, Anhui, 230031, PR China; 6 Department of Obstetrics and Gynecology, The First Affiliated Hospital of USTC, Division of Life Sciences and Medicine, Center for Advanced Interdisciplinary Science and Biomedicine of IHM, University of Science and Technology of China, Hefei, Anhui, 230001, PR China; 7 Institute of Health and Medicine, Hefei Comprehensive National Science Center, Hefei, Anhui, 231137, PR China; 8 Biomedical Sciences and Health Laboratory of Anhui Province, University of Science and Technology of China, Hefei, Anhui, 230027, PR China; 9 Clinical Research Hospital of Chinese Academy of Sciences (Hefei), University of Science and Technology of China, Hefei, Anhui, 230001, PR China

**Keywords:** 16S rRNA, comedones, inflammation, skin lesion, structure

## Abstract

Acne vulgaris is a common skin condition marked by the formation of comedones, papules, pustules and nodules, with its underlying causes still not fully understood. This study explores the impact of microbial dysbiosis and virulence factors on acne development. Through high-throughput 16S rRNA sequencing, we identified significant disruptions in the skin microbiome, particularly in comedones. Key virulence factors of *Cutibacterium acnes*, known as Christie–Atkins–Munch-Peterson (CAMP) factors, were assessed using both *in vitro* and *in vivo* models. Among these, CAMP2 and CAMP5 demonstrated the highest inflammatory and haemolytic activities in keratinocytes. Topical anti-IL-8 treatment in a murine model effectively reduced inflammation and suppressed CAMP expression. Structural analysis of CAMP3 uncovered distinct pathogenic features that, alongside CAMP5, were found to aggravate acne-like inflammation and sebaceous gland atrophy. These findings advance our understanding of the interplay between microbial dysbiosis and CAMP factors in acne pathogenesis, offering potential avenues for therapeutic intervention.

**Impact Statement**

Acne vulgaris is a prevalent skin disorder characterized by inflammation and structural changes in the skin, yet the precise role of microbial dysbiosis and virulence factors in its pathogenesis remains incompletely understood. This study provides critical insights into acne pathogenesis by uncovering the role of *Cutibacterium acnes* CAMP factors in disease progression. By integrating high-throughput microbiome analysis with cellular assays and animal models, the research highlights how specific CAMP factors, particularly CAMP2 and CAMP5, drive inflammation and tissue damage. The study further compares the pathogenicity of different CAMP factors, identifying the unique pathogenic features of CAMP3 and demonstrating the therapeutic potential of anti-IL-8 treatment. These findings refine the current view of acne pathogenesis by linking microbial dysbiosis to immune activation and offer a rationale for the development of targeted interventions to alleviate inflammation and tissue remodelling in acne-prone skin.

## Data Summary

The authors confirm that all supporting data and protocols have been provided within the article or through supplementary data files. The coordinates and structural factors of *Cutibacterium acnes* CAMP factor 3 have been deposited in the Protein Data Bank with accession code 9KX0 (https://www.rcsb.org/structure/unreleased/9KX0). Microbiome sequencing data were generated to profile the cutaneous microbial composition across different clinical groups. The raw sequencing reads have been deposited in the NCBI Sequence Read Archive (SRA) under BioProject accession number PRJNA1209331, with individual sample accession numbers SRR31979421 to SRR31979477.

## Introduction

Acne vulgaris is a widespread chronic inflammatory skin disorder, affecting ~9.4% of the population worldwide [1]. It is marked by the development of comedones and a range of inflammatory lesions, including papules, pustules, nodules and cysts [2,3]. The skin microbiota plays a key role in preserving its balance [4]. In acne, the equilibrium is disrupted by an overabundance of *Cutibacterium acnes (C. acnes)*, which reshapes the cutaneous niche and precipitates immune dysregulation and inflammation [5]. *C. acnes* has been identified as a predominant species within sebaceous glands, implicating it in the formation of early comedones and the progression to inflammatory acne lesions [6,8]. Variations in the skin microbiota composition and the relative prevalence of *C. acnes* across different ethnic groups suggest potential links to acne subtypes, indicating the necessity for a deeper investigation into these disparities [9].

Recent advancements have shed light on the secretion of Christie–Atkins–Munch-Peterson (CAMP) homologous proteins by *C. acnes* [10,11]. These proteins are capable of forming membrane pores and degrading host tissues, suggesting their involvement in acne pathogenesis [12]. While elevated levels of CAMP factors and inflammatory cytokines have been observed in acne lesions, the specific contributions of CAMP proteins to disease pathogenesis remain incompletely understood, underscoring the need for further mechanistic investigation [13,14].

In this study, we delved into the structure and function of *C. acnes* CAMP factors, as well as their haemolytic activity and inflammatory potential. Through a comparative analysis, we aimed to uncover the virulence mechanisms of these factors in acne pathogenesis and explore their potential as therapeutic targets.

## Methods

### Recruitment of participants and specimen collection

Participants were enrolled from the Dermatology Clinic of the First Affiliated Hospital, University of Science and Technology of China (USTC), during the period from June to October 2023. The study was approved by the USTC Biomedical Ethics Committee (Approval No. 2023KY130). Eligible participants included individuals aged 18–45 with facial comedonal or inflammatory acne lesions, without any concurrent skin conditions or recent (within the past 6 months) treatments with isotretinoin or antibiotics. Age-matched controls, free from any skin diseases and medication use during the same period, were also included. Informed written consent was obtained from all of the participants. Tissue collection involved facial disinfection, followed by lesion extraction or puncture. The contents were transferred onto sterile swabs and stored in PBS at −80 °C [15]. The samples were later delivered to Biotree Biotech Co., Ltd. for metabolomic examination.

### Genome DNA extraction and 16S rRNA sequencing

Genomic DNA was isolated employing an enhanced cetyltrimethylammonium bromide protocol to ensure high-quality yields. The amplified PCR products were analysed on a 2% agarose gel to confirm their integrity and then purified using AMPure XT magnetic beads for further processing. Sequencing libraries were constructed following standard protocols and subsequently processed on the NovaSeq PE250 platform to obtain high-resolution 16S rRNA sequencing data. All sequencing data have been deposited in the NCBI Sequence Read Archive under BioProject accession number PRJNA1209331 (SRA accession numbers SRR31979421–SRR31979477).

### Analysis of 16S rRNA sequencing

The sequencing process was conducted using the Illumina NovaSeq platform. Sequences were processed using fqtrim, Vsearch and DADA2 for feature tables. Alpha and beta diversity metrics were calculated using QIIME2. Taxonomic classification was annotated using the SILVA database [16]. Additional graphical representations were generated with the R package (v3.5.2).

### Meta-analysis

A comprehensive meta-analysis was carried out in accordance with the Preferred Reporting Items for Systematic Reviews and Meta-Analyses (PRISMA) guidelines. Studies available up to 13 December 2023 were thoroughly searched across PubMed, Embase, Cochrane Library and Web of Science databases. The search incorporated the terms ‘acne’ and ‘*Cutibacterium acnes*’ or ‘*Propionibacterium acnes*’. Study selection, data extraction and outcome analysis were conducted according to protocols described previously [17].

### Animal studies

Institute of Cancer Research (ICR) mice were procured from Shanghai Model Organisms Center, China, and housed in sterile, specific pathogen-free conditions for the duration of the study. All experimental protocols involving animals were reviewed and approved by the Experimental Animal Management Committee of USTC (Approval No. USTCACUC26080122046).

Eight-week-old male ICR mice were randomly assigned to different groups: the control group was administered 50 µl of PBS, the * C. acnes* group received 50 µl of *C. acnes* suspension (1×10^7^ c.f.u. ml^−1^) and the anti-IL-8-treated group received both *C. acnes* suspension (1×10^7^ c.f.u. ml^−1^) and topical anti-IL-8 treatment. The anti-IL-8 treatment contained a commercially available Mouse Monoclonal Antibody against Human Interleukin-8 Cream (Abcream, Yes Biotech Laboratories Ltd., NMPA registration number: S20030093) at a concentration of 0.045 mg g^−1^, formulated in a neutral hydrophilic cream base. The *C. acnes* suspension was intradermally injected into the central portion of the right ear of ICR mice to induce acne-like dermatitis. Mice in the anti-IL-8-treated group received topical application of Mouse Monoclonal Antibody against Human Interleukin-8 Cream onto their ears 30 min after *C. acnes* application, once daily. In another set of experiments, 8-week-old male ICR mice were injected with CAMP2–5 proteins (8 mg ml^−1^ in 200 µl) subcutaneously on the back, once daily for two consecutive days. Fresh ear and back skin tissues were collected for RNA analysis.

For histological analysis, sections preserved in formalin and embedded in paraffin were stained using haematoxylin and eosin (H and E). Immunofluorescence staining was performed using PPAR*γ* (Cell Signaling, 2435T) and K10 (Abcam, ab76318) antibodies, as described previously [18].

### Quantitative real-time PCR (RT-qPCR)

Total RNA (200 ng) was reverse transcribed into cDNA using the PrimeScript RT Reagent Kit (TaKaRa, RR037A), following the manufacturer’s guidelines. RNA concentration was measured with a NanoDrop 2000 (Thermo Fisher Scientific Inc). The cDNA was then analysed by RT-qPCR using TB Green Premix Ex Taq II (Tli RNaseH Plus) (TaKaRa, RR820A). Gene expression levels were calculated using the comparative ΔΔCt method [19]. Fig. 5f was generated as a heatmap using the online platform https://www.bioinformatics.com.cn, a web-based tool for data analysis and visualization. The primer sequences used in this study are provided in Table S1 (available in the online Supplementary Material).

### Expression, purification and crystallization of CAMP factors

*C. acnes* CAMP factor proteins 1–5 (NCBI accession code AAT83089.1, AAT82444.1, PHJ26407.1, AAT82980.1 and AAT82947.1) were expressed using the pGEX vector in *Escherichia coli*, with an MBP tag and a TEV protease cleavage site, following the established protocols [20,21].

The purified proteins were concentrated to 20 mg ml^−1^ using Amicon centrifugal concentrators (Millipore). Crystallization was performed using the hanging drop vapour diffusion method in a solution containing 26% PEG3350, pH 5.0. Crystals of CAMP factor 3 were observed after 3 weeks at 18 °C. To protect the crystals for X-ray analysis, they were briefly washed and flash cooled in liquid nitrogen using a solution containing 15% ethylene glycol.

### Cultivation of *C. acnes*

*C. acnes* ATCC 6919 (American Type Culture Collection, Manassas, VA) was cultured on ATCC Medium 2107 (Biofeng, China) under anaerobic conditions using AnaeroPack-Anaero (Mitsubishi, Japan) at 37 °C. Bacteria were harvested, washed and resuspended in PBS for experiments. Heat-killed *C. acnes* was prepared by incubating cultures at 80 °C for 30 min.

### Cell culture and stimulation

Human keratinocyte cells (HaCaT, CL-0064, Procell, Wuhan, China) were maintained in Dulbecco’s Modified Eagle Medium (Gibco), supplemented with 15% FBS (Gibco) and 1% penicillin-streptomycin solution (Procell, China) and incubated at 37 °C with 5% CO_2_.

For stimulation, HaCaT cells were seeded in 24-well plates and treated with varying concentrations of CAMP factors (10, 5, 0.5 and 0.05 µM) for 36 h [10,22]. The treatment duration was selected based on preliminary CCK-8 assays (Juyan Biotechnology Co., Ltd., Hefei, China), which demonstrated a strong inflammatory response without significant cytotoxicity at this time point (Fig. S3a) [23,24]. The levels of IL-6 and IL-8 in the culture supernatants were quantified using ELISA kits (Absin, Shanghai, China). For apoptosis analysis, HaCaT cells were plated in a 12-well plate and treated with 10 mM CAMP factors for 36 h. Apoptosis was measured using the Annexin V-PE/7-AAD Apoptosis Detection Kit (KeyGEN BioTECH, China).

### X-ray diffraction, structure determination and refinement

X-ray diffraction data were collected at GM/CA-CAT at the Shanghai Synchrotron Radiation Facility (SSRF). The structure was determined using molecular replacement with the *C. acnes* CAMP model from the Protein Data Bank (PDB ID: 9KX0). Refinement was carried out through iterative cycles of model building in Coot, followed by automatic refinement with Phenix.refine. Structural visualizations were generated using PyMOL (pymol.org).

### Haemolysis assay

Sheep red blood cells (SRBCs, HQ80073-004, Hongquan Biotech, Guangzhou, China) were used for haemolysis assay as previously described [21,25]. Briefly, SRBCs were pre-sensitized with staphylococcal sphingomyelinase (50 mU, Sigma) at room temperature for 30 min to facilitate pore formation. The pre-treated SRBCs were then washed and resuspended in HEPES-buffered saline (10 mM HEPES, 150 mM NaCl, 10 mM MgCl_2_ and pH 6.5). Each CAMP factor (CAMP2–CAMP5) was added at a final concentration of 1 µg ml^−1^ and incubated with the SRBC suspension at 37 °C. Haemolytic activity was assessed by measuring the OD at 650 nm (OD₆₅₀) at 1-h intervals. Haemolysis was quantified based on the release of haemoglobin into the supernatant. Each experimental condition was tested in triplicate.

### Statistical analysis

Data were expressed as means±sd. Comparisons between two groups were performed using Student’s t-test, while two-way ANOVA followed by Bonferroni post hoc testing was applied for multiple group comparisons. Statistical significance was defined as *P<0.05, **P<0.01, ***P<0.001 and ****P<0.0001.

## Results

### Microbial dysbiosis is evident within the contents across different types of acne lesions

**Fig. 1.**
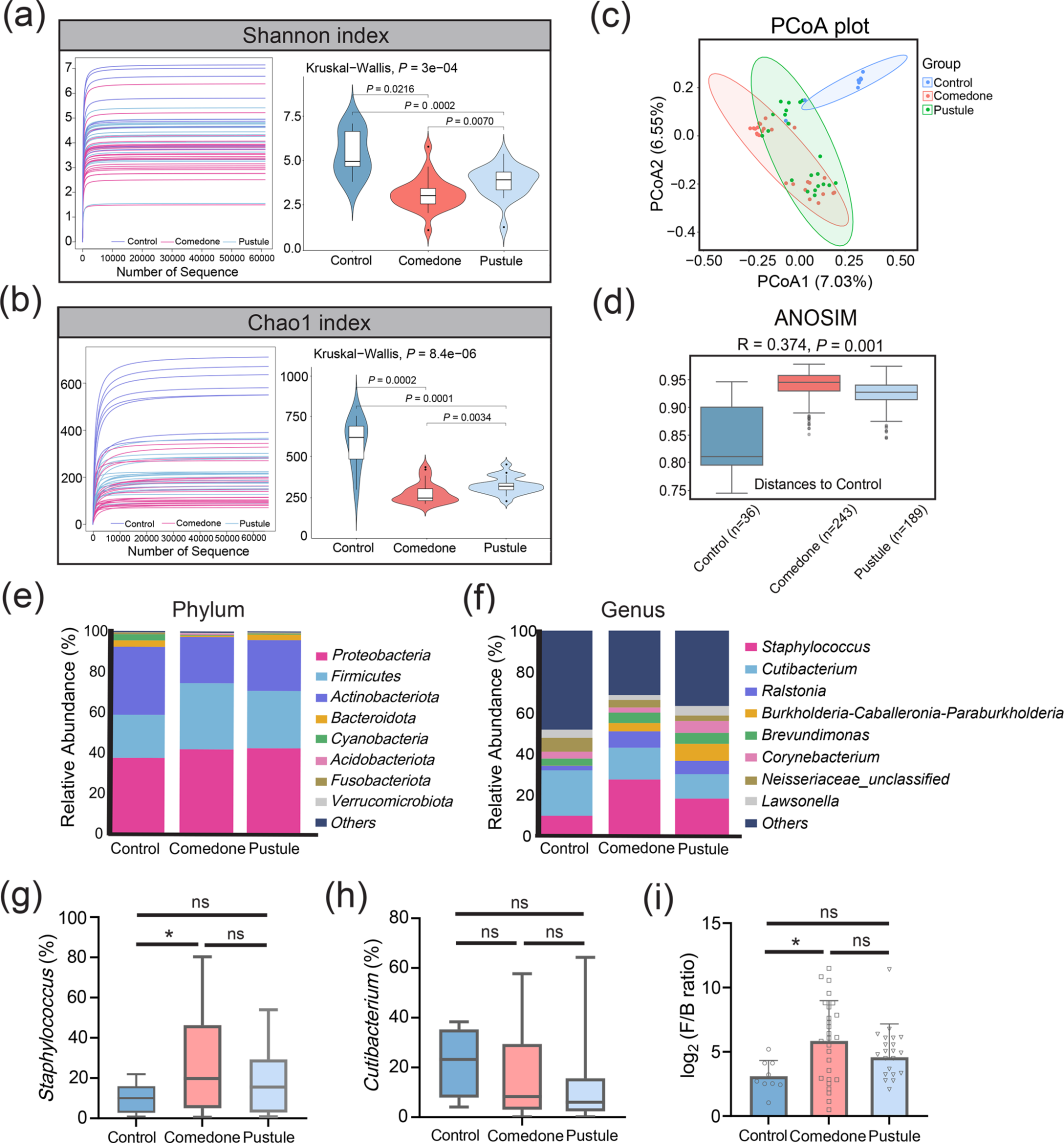
The study characterizes skin microbiota richness and diversity in acne lesions using 16S rRNA gene sequencing. (a, b) The assessment of skin microbiota richness and diversity in acne lesion contents using Shannon and Chao1 indices, with statistical analysis performed using the Kruskal–Wallis test followed by post hoc analysis to determine the significance of any observed differences. (c) PCoA based on the Jaccard dissimilarity coefficient is applied to illustrate the distinct microbial community structures among the comedone, pustule and control groups. (d) The ANOSIM test confirms significant microbial community dissimilarity among the groups (*R*=0.374, *P=0.001). (e, f) Comparative analysis of bacterial phylum and genus across comedone (*n*=27), pustule (*n*=21) and control groups (*n*=9). (g, h) Genus-level analysis is performed to assess the abundance of *Staphylococcus* and *Cutibacterium* (*C. acnes*) among the acne lesion groups (*P<0.05). (i) The *Firmicutes* to *Bacteroidetes* (F/B) ratio is calculated and compared among groups to suggest a potential link to an inflammatory state, as determined by sequencing data (*P<0.05).

In order to explore the microbial composition across various acne lesion types, we curated a dataset comprising 27 comedone samples, 21 pustule samples, and 9 normal skin tissue controls. Leveraging 16S rRNA gene sequencing techniques, we analysed the microbial content of these samples. Alpha diversity analysis revealed significant differences in microbial richness and diversity between the control, comedone and pustule groups. Furthermore, the comedone group exhibited lower diversity compared with the pustule group, as evidenced by reduced Shannon (*P*=0.0070) and Chao1 (*P*=0.0034) indexes. (Fig. 1a, b). Principal coordinate analysis (PCoA) based on the Jaccard dissimilarity further highlighted these differences (Fig. 1c), and analysis of similarities (ANOSIM) confirmed significant variability among the groups (*R*=0.374, *P*=0.001, Fig. 1d). These findings suggest the presence of microbial dysbiosis in acne patients, particularly within the comedonal acnes.

We next examined the taxonomic profiles at the phylum and genus levels. *Proteobacteria*, *Firmicutes*, *Actinobacteriota* and *Bacteroidota* were the predominant phyla, with varying relative abundances across the groups (Fig. 1e). Specifically, *Proteobacteria* accounted for 37.3%, 41.6% and 42.2% of the microbial community in the control, comedone and pustule groups, respectively, while *Firmicutes* were more abundant in the comedone (32.6%) and pustule (28.3%) groups compared with the control (21.3%). Notably, the relative abundance of *Staphylococcus* increased significantly in the comedone group (27.3%), whereas *C. acnes* showed no significant differences among the groups (Fig. 1f–h). Importantly, the *Staphylococcus*/*C. acnes* (S/C) ratio was substantially elevated in acne lesions, supporting the notion that acne pathogenesis is more closely associated with microbial dysbiosis rather than simple overgrowth of *C. acnes* alone [8] (Fig. S1).

Additionally, the *Firmicutes* to *Bacteroidetes* (F/B) ratio was significantly altered in the comedone group (*P*=0.016, Fig. 1i). Although the F/B ratio has been extensively studied in the gut microbiome where increases are associated with conditions such as psoriasis, metabolic syndrome and inflammatory bowel disease, emerging evidence suggests that similar microbial shifts may also reflect early dysbiosis in the skin [26]. These parallels, particularly in relation to epithelial barrier dysfunction and immune activation, imply that alterations in the skin F/B ratio may serve as an early marker of acne development [27]. These findings imply that microbial dysbiosis may precede the inflammatory stages of acne, with comedones potentially serving as a precursor to more severe lesions.

To further validate these findings, we performed a meta-analysis of skin microbiota data from 18 studies, including 102 control samples and 401 acne samples (Table S2) [6,9, 28,43]. The analysis confirmed that *C. acnes* was the dominant micro-organism in acne lesions, accounting for 49% (range: 27%–71%) of all clones (Fig. S2a). Although no significant differences in *C. acnes* abundance were observed between the acne and control groups [standard mean difference (SMD)=−0.03 (−0.57 to 0.51), Fig. S2b and c], a reduction in diversity within *C. acnes* strains was identified as a potential factor in acne onset and progression. Healthy skin can tolerate *C. acnes* presence, but acne promotes an interaction between *C. acnes* and follicular cells, triggering inflammation and exacerbating lesion development [44]. Furthermore, recent studies suggest that *C. acnes* may enhance its virulence in synergy with CAMP factors secreted by acne-associated strains, warranting further investigation [23].

### The skin microbiota emerges as promising predictive indicators for different acne susceptibility levels

**Fig. 2.**
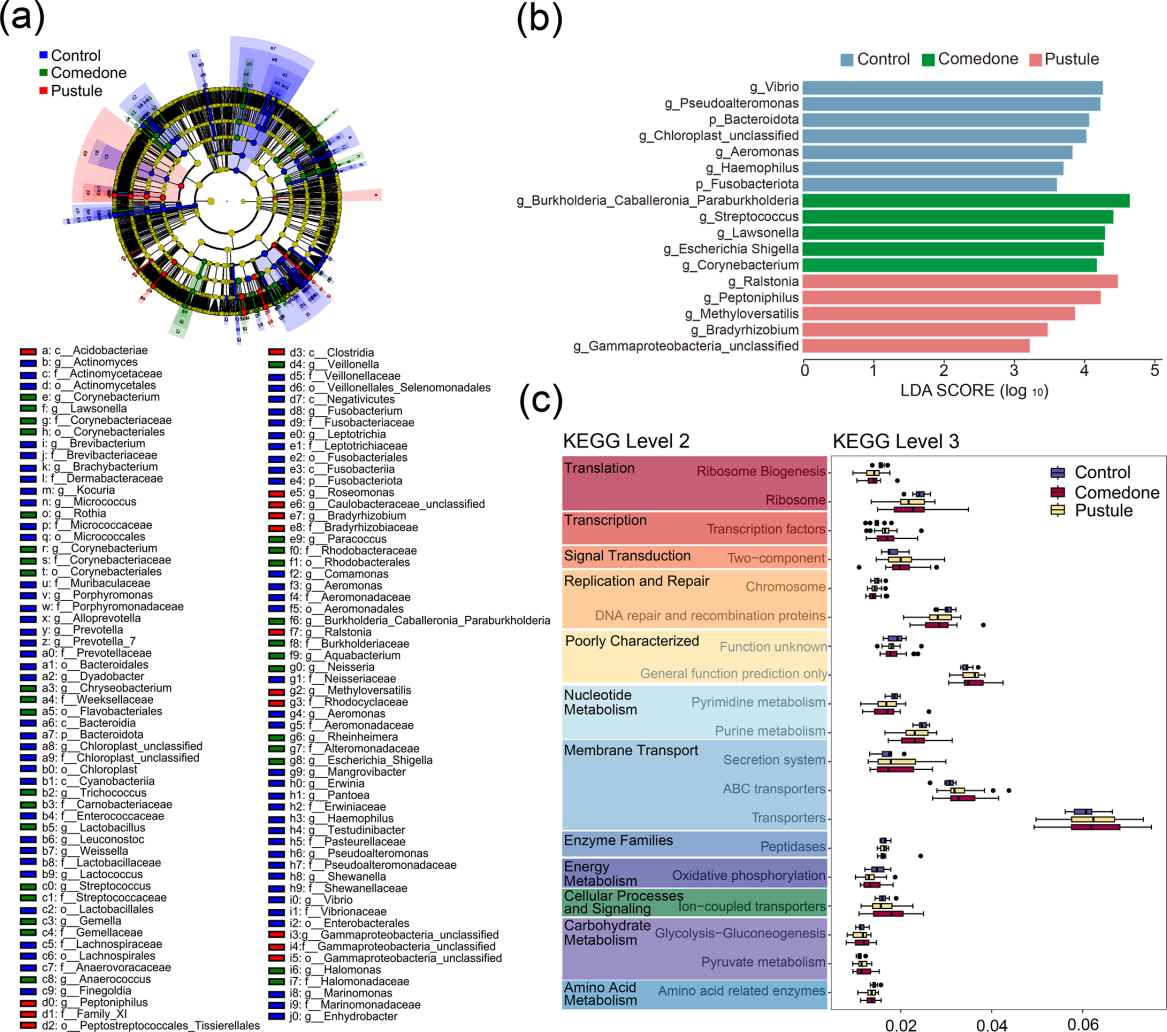
Diagnostic biomarkers and functional analysis prediction are identified for acne in the skin microbiota. (a) Utilizing LEfSe analysis, we identified the most characteristic taxonomic groups at the phylum and genus levels of the OTU table. (b) The top five biomarkers at the phylum and genus levels were selected, which have the potential to serve as microbial signatures distinguishing acne from normal samples. (c) Through PICRUSt analysis, we further explored the metabolic potential of microbial communities within acne lesions, identifying 5 significant KEGG level 2 pathways and 20 level 3 pathways. These findings highlight the potential utility of exploiting microbial differences for acne classification and therapeutic interventions.

The skin microbiota holds promise as a predictive indicator for acne susceptibility [39]. Using linear discriminant analysis (LDA) effect size (LEfSe) and LDA, we identified microbial signatures associated with different acne types. At both the phylum and genus levels, taxonomic differences were apparent, with the control group exhibiting the highest number of discriminative biomarkers (Fig. 2a). Among these, *Streptococcus* emerged as a key biomarker for acne (Fig. 2b). This genus has previously been implicated in conditions such as atopic dermatitis and tetracycline resistance [42]. In acne patients undergoing tetracycline treatment, a reduction in *C. acnes* is often accompanied by an increased abundance of *Streptococcus*, suggesting a potential contributory role in disease progression [45]. These findings emphasize the necessity of targeting microbial community balance, rather than focusing exclusively on *C. acnes*, in the development of effective acne therapies [6,30, 35, 40]. Low-abundance taxa of environmental or gut origin (e.g. *Chloroplast* and *Escherichia-Shigella*) were also detected. These likely reflect external contamination or sample handling artefacts and are not indicative of a functional role in acne. However, recent studies have reported gut-associated bacteria in non-intestinal sites during inflammation, though this remains under debate [46].

Additionally, using Phylogenetic Investigation of Communities by Reconstruction of Unobserved States (PICRUSt) analysis, we predicted differences in metabolic potential across acne lesions, identifying significant Kyoto Encyclopedia of Genes and Genomes (KEGG) pathways. The pustule group exhibited increased activity in pathways related to membrane transport, cellular processes, signalling and carbohydrate metabolism (Fig. 2c). These findings suggest that microbiota differences can aid in acne classification and guide more effective therapeutic strategies.

### *C. acnes* CAMP factors exhibit varying degrees of toxicity and haemolytic capability

**Fig. 3.**
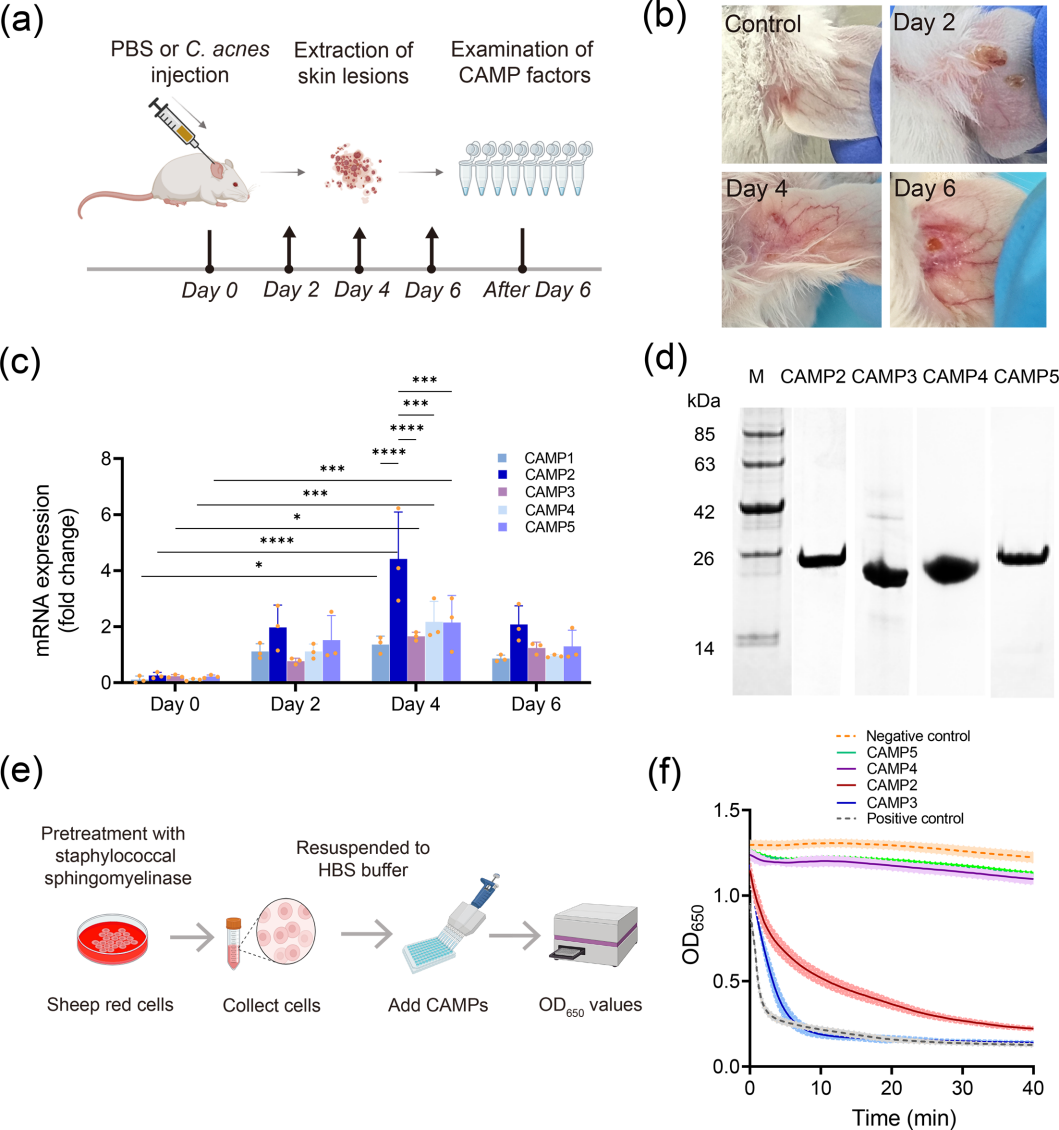
Haemolytic and toxic properties vary among *C. acnes* CAMP factors. (a) Schematic timeline of intradermal injection of *C. acnes* into the ears of ICR mice (*n*=3 per time point), followed by collection of skin lesion samples on days 0, 2, 4 and 6. (b) Visual representation of induced swelling and cutaneous erythema at various time points in the injected ear. (c) Quantitative RT-qPCR showed the mRNA expression levels of CAMP factors 1–5 in skin lesions over 6 days, normalized to expression levels at day 0. Data are presented as mean±sem (*n*=3). Statistical analysis was performed using one-way ANOVA (****P<0.001, ***P<0.001, **P<0.01, *P<0.05). (d) SDS-PAGE gel electrophoresis showed purified recombinant CAMP2–5 proteins, with molecular weight markers indicated in kilodaltons. (e) Haemolytic activity of CAMP factors 2–5 was evaluated using SRBCs pre-sensitized with staphylococcal sphingomyelinase (50 mU, 30 min, room temperature). SRBCs were suspended in HEPES-buffered saline (10 mM HEPES, 150 mM NaCl, 10 mM MgCl_2_ and pH 6.5) and incubated with 1 μg ml^−1^ of each CAMP factor. Haemolysis was monitored by measuring OD at 650 nm (OD_650_) at 1-h intervals. Each condition was tested in triplicate. (f) Time-course changes in OD_650_ reflect the haemolytic activity of CAMP factors. Graphs were generated using GraphPad Prism.

In a previous investigation, we identified five nucleotide sequences encoding the putative toxin CAMP factors within the *C. acnes* genome [21]. To gauge the expression levels of these factors, we intradermally injected *C. acnes* bacteria into the ears of ICR mice (Fig. 3a). This procedure induced swelling and severe erythema in the injected ear (Fig. 3b). All CAMP factors were detectable in the lesions, with CAMP2 showing the highest expression on the second day (Fig. 3c). These results suggest that the CAMP factors play a significant role in skin inflammation, with *C. acnes* increasing the expression of CAMP1–5.

To further explore the functional roles of these CAMP factors in *C. acnes* pathogenesis, we expressed the signal peptide-truncated coding sequences of CAMP factors in *E. coli* (Table S3) [47]. Since the functionality of CAMP1 has already been well documented [47], we focused on investigating the properties of CAMP2–5. Following Ni-NTA purification, we successfully isolated highly pure forms of four *C. acnes* CAMP proteins 2–5 (Fig. 3d). Known for their involvement in the CAMP reaction [48], we examined their haemolytic activity on red blood cells pretreated with sphingomyelinase, at the pH of acne-affected skin (~6.5) [49] (Fig. 3e). Our findings revealed that CAMP2 and CAMP3 exhibited strong haemolytic activity, while CAMP4 and CAMP5 did not (Fig. 3f). These results highlight the varying cytotoxicity and haemolytic potential of the CAMP factors, suggesting distinct roles in acne pathogenesis.

### CAMP proteins promote inflammatory cytokine production and apoptosis in keratinocytes

**Fig. 4.**
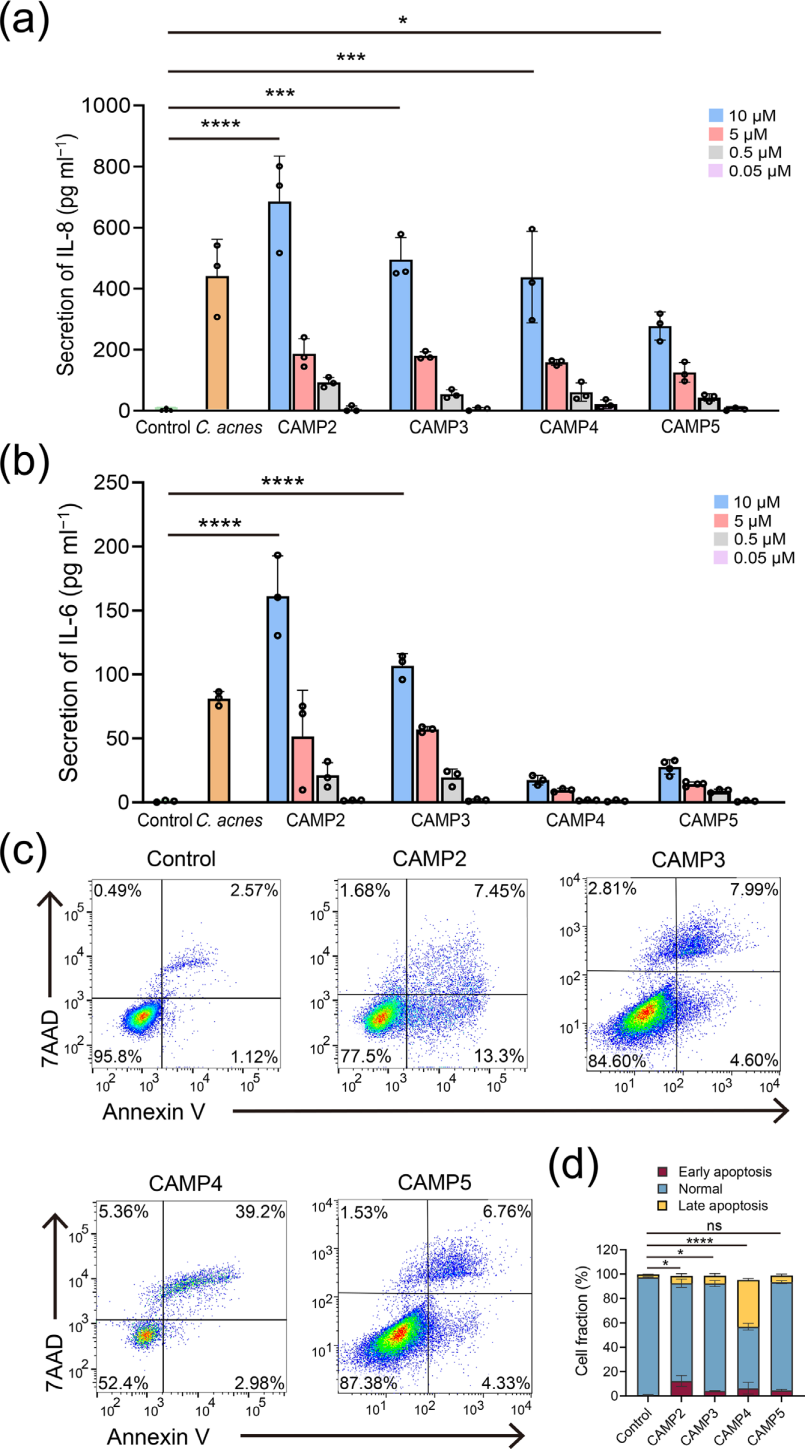
CAMP proteins induce inflammatory cytokine production and apoptosis in keratinocytes. (a) Dose-dependent induction of IL-8 by epidermal keratinocytes upon stimulation with different concentrations of CAMP proteins. (b) IL-6 secretion levels in response to CAMP protein stimulation. Control and *C. acnes* treatments are shown for comparison. Statistical significance was determined by one-way ANOVA (*n*=3, ****P<0.0001, ***P<0.001, *P<0.05). (c) Flow cytometry analysis of keratinocyte apoptosis after treatment with 10 μM of CAMP proteins for 24 h using Annexin V and 7-AAD staining. (d) Quantification of early and late apoptotic fractions across all treatment conditions, based on flow cytometry (*n*=3, ****P<0.0001, *P<0.05; ns, not significant).

In acne pathogenesis, the upregulation of Th17-related cytokines such as IL-8, IL-1*β*, IL-6 and TGF-*β* is well documented [50,51]. This study examined whether CAMP proteins directly interact with keratinocytes to induce inflammation. Our results showed that stimulation with CAMP proteins led to a dose-dependent increase in IL-8 production, with CAMP2 and CAMP3 exhibiting stronger effects on IL-6 expression (Fig. 4a, b). No significant changes were observed in IL-1*β*, IL-2, IL-4, IL-10, IL-12 or IFN-*γ* production (Fig. S3b).

**Fig. 5.**
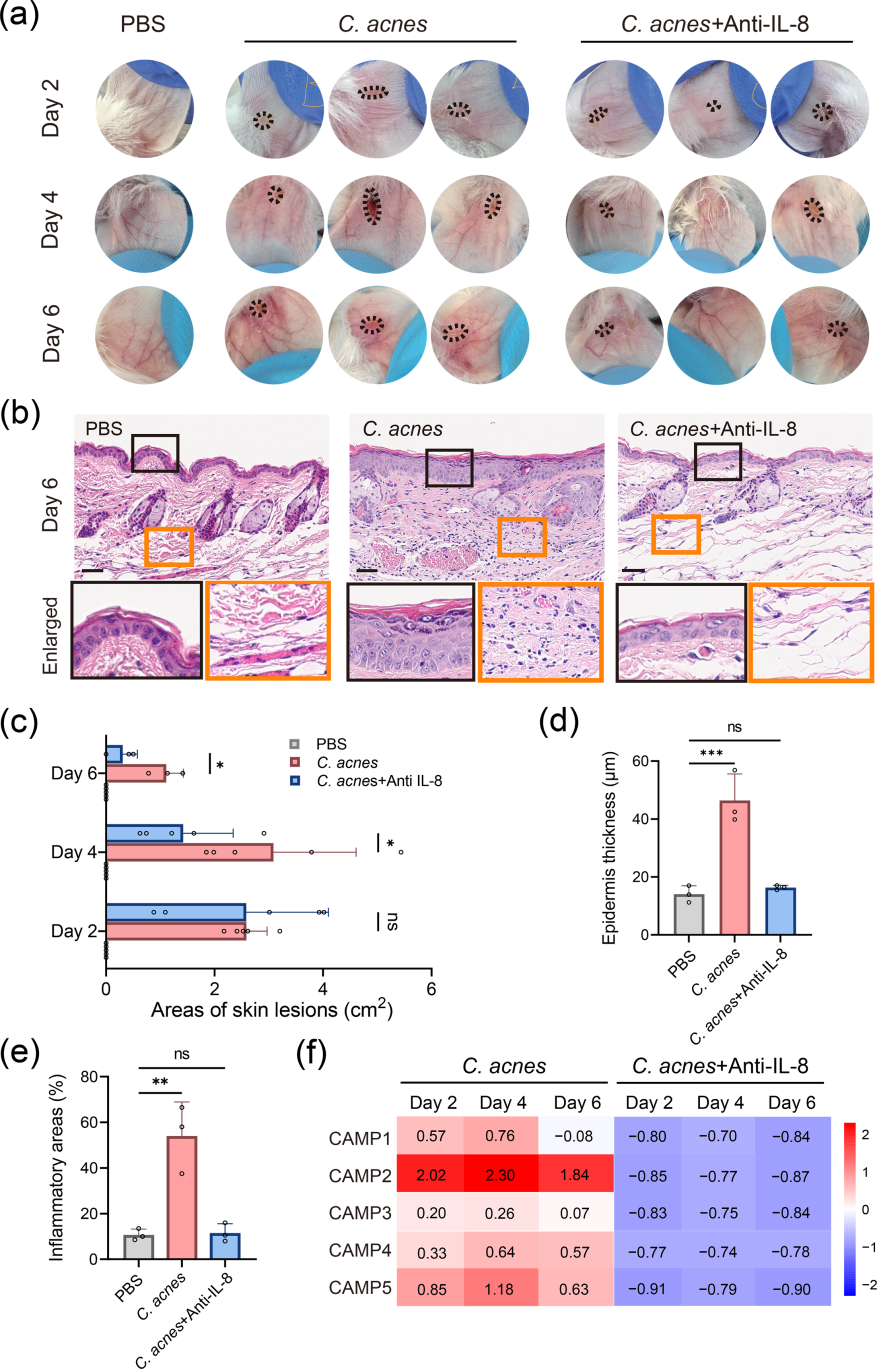
Topical anti-IL-8 medication ameliorates acne-like dermatitis and downregulates CAMP factor expression. (a) Macroscopic assessment of ear inflammation in mice treated with PBS, *C. acnes* and a combination of *C. acnes* with anti-IL-8 at various time points (*n*=3). (b) Histological analysis of ear sections from mice treated with PBS, *C. acnes* and *C. acnes* with anti-IL-8 (scale bar: 50 µm). (c) Quantification of skin lesion areas in square centimetres for each treatment group (*P<0.05; ns, not significant). (d, e) Measurement of skin thickness (micrometre) and inflammatory area (per cent) from histological images (***P<0.001, **P<0.01; ns, not significant). (f) Heatmap of relative mRNA expression levels of CAMP factors 1–5 in ear tissues of *C. acnes*-injected mice treated with or without anti-IL-8 cream. Expression levels were quantified by RT-qPCR and calculated relative to baseline expression at day 0. Heatmap values represent mean fold change from three biological replicates. Data visualization was performed using the online platformBioinformatics.com.cn.

To further explore the cytotoxic effects of these CAMP proteins, we assessed their impact on keratinocyte viability *in vitro* (Fig. 4c). As shown in Fig. 4d, treatment with CAMP2 resulted in a significant increase in early apoptotic cells. CAMP3, CAMP4 and CAMP5 induced comparable levels of cytotoxicity. Additionally, all CAMP-treated groups exhibited an increased proportion of necrotic cells, with CAMP4 inducing the most pronounced reduction in total cell count, accompanied by the highest level of necrosis. These findings indicate that *C. acnes* CAMP factors contribute to both inflammation and apoptosis in keratinocytes, with IL-8 playing a central role in acne-related inflammation. Given its involvement, IL-8 could be a promising target for therapeutic interventions in acne treatment [44,52].

### Topical anti-IL-8 medication ameliorates acne-like skin dermatitis and downregulates CAMP factors

To evaluate the effects of IL-8 inhibition on acne-like skin inflammation, we topically applied a combination of *C. acnes* and a monoclonal antibody targeting human IL-8 (anti-IL-8) to mice with acne-like dermatitis. Initially, no significant changes were observed. However, by day 4, the condition in the *C. acnes*-treated group worsened significantly. In contrast, mice treated with both *C. acnes* and anti-IL-8 showed marked improvement, with reduced redness and skin lesion areas (Fig. 5a).

Histological analysis revealed reduced epidermal thickness and inflammatory cell infiltration in the anti-IL-8 group (Fig. 5b–e). Additionally, CAMP factor expression was significantly lower in the anti-IL-8-treated mice (Fig. 5f). These results suggest that the topical application of anti-IL-8 effectively alleviates acne-like skin dermatitis, potentially by downregulating CAMP factor expression. These findings suggest that IL-8 inhibition effectively mitigates acne-like dermatitis, potentially by downregulating CAMP factor expression, highlighting IL-8 and CAMP proteins as promising therapeutic targets in acne treatment.

### CAMP factors play a role in inducing acne-like lesions in mice

**Fig. 6.**
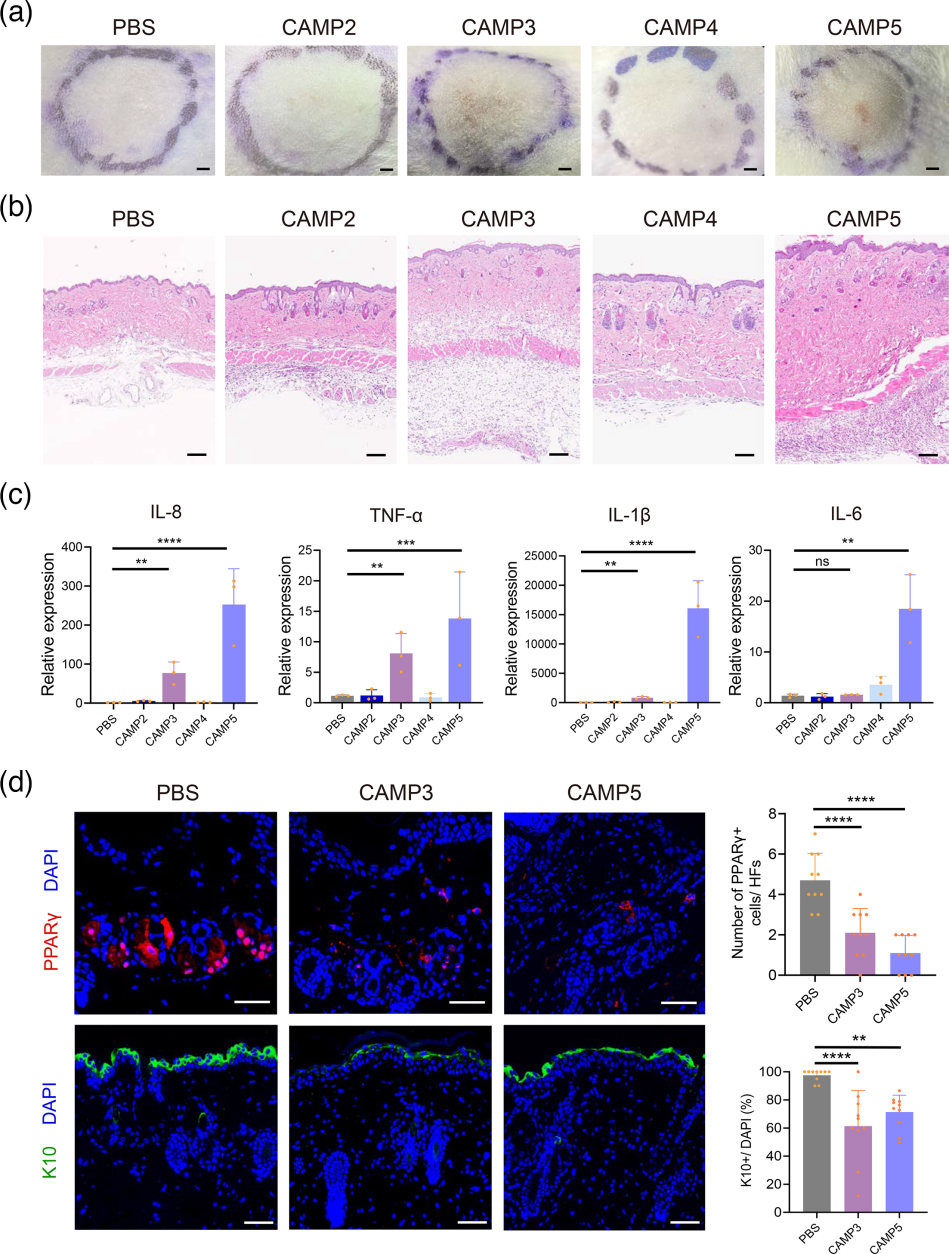
CAMP factors trigger acne-like lesions in mice. (a, b) Representative overviews (scale bar: 0.2 cm) and HE staining (scale bar: 100 µm) of dorsal skin treated with CAMP proteins or PBS (*n*=4). (c) RT-qPCR analysis of mRNA levels of acne-characteristic factors. Statistical significance was calculated using one-way ANOVA (*n*=3, ****P<0.0001, ***P<0.001, **P<0.01; ns, not significant). (d) Immunofluorescence staining and quantification of PPAR*γ* and K10 expression in mouse dorsal skin (scale bar: 100 µm; *n*=3; ****P<0.0001, ***P<0.001, **P<0.01).

To further explore the *in vivo* effects of CAMP proteins, we injected *C. acnes* CAMP factors 2–5 into the dorsal skin of mice once daily for two consecutive days. Our observations revealed that CAMP3 induced erythema and scaling on the dorsal skin, while CAMP5 led to the formation of pronounced subcutaneous nodules. In contrast, CAMP2 and CAMP4 did not produce noticeable skin changes at the same dosage (Fig. 6a, b).

The analysis of inflammatory markers in the skin showed increased levels of IL-8, TNF-*α*, IL-1*β* and IL-6, consistent with the observed tissue inflammation (Fig. 6c). Notably, IL-1*β* expression was markedly upregulated *in vivo* following CAMP5 treatment, a response not recapitulated *in vitro* (Fig. S3b). This discrepancy likely reflects the complexity of the *in vivo* immune microenvironment [53]. Resident immune cells such as macrophages and dendritic cells, which are absent or underrepresented in keratinocyte monocultures, may substantially contribute to IL-1*β* production [53,54]. Furthermore, CAMP5 may engage inflammasome pathways *in vivo* that depend on intercellular interactions or the presence of damage-associated molecular patterns released during tissue injury, mechanisms that are not readily replicated *in vitro* [55].

Immunofluorescence staining further revealed decreased expression of the sebaceous gland markers PPAR*γ* in pilosebaceous units and epidermal differentiation marker K10 in suprabasal layers (Fig. 6d). These findings suggest that CAMP3 and CAMP5 may contribute to skin inflammation and potentially affect sebaceous gland function. However, further investigation is warranted to clarify the underlying mechanisms and confirm their specific roles in the pathogenesis of acne-like lesions [56].

### Crystallization and structure determination of *C. acnes* CAMP factor 3

**Fig. 7.**
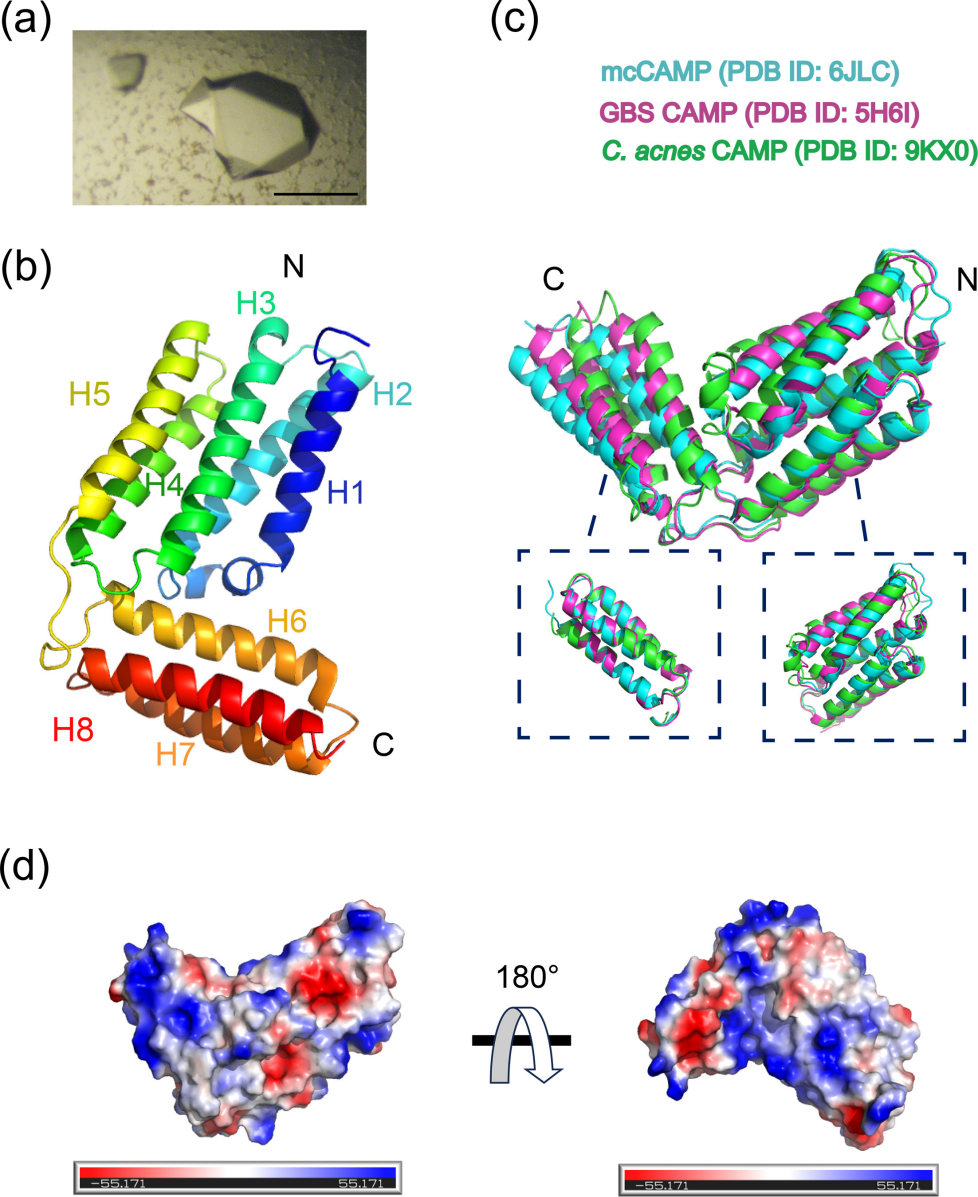
Crystallization and structure determination of *C. acnes* CAMP factor 3. (a) Single crystals of CAMP3 (scale bar: 100 µm). (b) The overall structure of CAMP3. The resolution of the structure is 2.43 Å (PDB ID: 9KX0). (c) Structural comparison of the *C. acnes* CAMP3 factor (green) with mcCAMP factor (blue) and GBS CAMP factor (red). (d) Electrostatic surface models of *C. acnes* CAMP3 factor display negatively charged regions in red, neutral regions in white and positively charged regions in blue.

To further analyse the molecular mechanism of *C. acnes* CAMP factors, we determined the crystal structure of CAMP3. Under acidic crystallization conditions (26% PEG3350, pH 5.0), long rod-shaped crystals of CAMP3 were formed (Fig. 7a). The crystals diffracted to 2.43 Å resolution and belonged to space group *P*4_1_22. The final refined model gave R/Rfree values of 24.31%/30.11% for all data to 2.43 Å resolution. The root-mean-square deviations (RMSDs) from ideal values were 0.0062 Å for bond lengths and 1.2773° for bond angles. The Ramachandran plot showed 96.85% of residues in favoured regions (Table S4). *C. acnes* CAMP factor 3 is an all-helical protein composed of two distinct structural modules: the N-terminal five-helix bundle (A44–A179) and the C-terminal three-helix bundle (V191–D260) (Fig. 7b). These domains are linked by a conserved loop containing the DLXXXDXAT motif, crucial for haemolytic activity.

Sequence alignment with CAMP factors from other species is shown in Fig. S4. The *C. acnes* CAMP factor 3 shares 37.55% and 35.32% sequence identity with the *Mobiluncus curtisii* (mc) CAMP factor and *Streptococcus agalactiae* (GBS) CAMP factor, respectively. Despite the low sequence identity, structural comparisons revealed significant similarity, with RMSD values of 2.79 and 2.38 Å. When comparing the C-terminal domain and N-terminal domain separately, the differences were smaller (RMSD: 0.446 and 0.600 Å; 1.665 and 1.426 Å), suggesting that the variations might be related to the angle differences (Fig. 7c). To examine the differing surface charge properties of mcCAMP and GBS CAMP factors, which may affect ligand binding and function, we generated electrostatic surface representations using PyMOL. Notably, both surfaces predominantly exhibited positive charge (Fig. 7d).

## Discussion

Preserving the integrity of the skin microbiome is paramount for fending off pathogens and adapting to environmental stressors [4]. Our study highlights that acne lesions, particularly comedones and pustules, display significant microbial deviations from healthy skin, with a notable reduction in bacterial diversity. This observation aligns with patterns seen in other dermatological disorders, such as atopic dermatitis and rosacea, thereby underscoring the broader relevance of microbiome health to dermatological conditions [57,59]. Interestingly, comedones showed a more pronounced decrease in microbial diversity than pustules, raising the possibility that dysbiosis may occur early in the course of lesion development [36]. While comedones do not always progress into pustules, this finding suggests that microbial imbalance may precede visible inflammation in some cases [60]. Therefore, interventions aimed at maintaining or restoring microbial balance during these early stages may offer a promising approach to reducing acne severity or preventing lesion progression [59].

While *C. acnes* play a central role in acne development, it also contributes to the cutaneous natural defence by outcompeting harmful bacteria [61]. Taking into account variables such as sampling sites, population demographics, gender and acne severity, we performed a meta-analysis of 18 studies investigating acne and the skin microbiota [5]. Our analysis showed no significant difference in *C. acnes* relative abundances, supporting the notion that acne is more related to microbial imbalance, particularly the ratio of *C. acnes* to *Staphylococcus*, rather than an overgrowth of *C. acnes* alone [8]. Mechanistically, studies have shown that *Staphylococcus β*-haemolysin can act synergistically with *C. acnes* to disrupt host cell membranes, facilitate tissue invasion and promote ceramide release, which may enable bacterial dissemination via a cell-to-cell mechanism [23,62]. Additionally, *Staphylococcus* can ferment endogenous carbon sources such as glycerol or glucose within acne lesions to produce short-chain fatty acids (SCFAs) [63]. Certain SCFAs have been shown to inhibit histone deacetylase activity, thereby amplifying TLR2- and TLR3-mediated cytokine responses and exacerbating cutaneous inflammation [64,65]. These findings suggest that microbial cross-talk between commensals and opportunistic pathogens may play a critical role in driving local inflammation and tissue damage in acne lesions.

In parallel, *Streptococcus* has also been identified as a significant biomarker for acne. Previous research indicates that *Streptococcus* plays an important role in both atopic dermatitis and tetracycline resistance [66]. For instance, in acne patients undergoing tetracycline treatment, *C. acnes* levels often decrease while *Streptococcus* abundance increases, suggesting a compensatory overgrowth that may contribute to lesion persistence or recurrence [67]. Moreover, chronic low-grade *Streptococcus* can persist within the lymphatic system, migrating from the liver to lymphatic tissues and potentially reaching the skin, which may lead to the development of cystic acne [68]. Collectively, these findings emphasize that acne pathogenesis is not solely attributable to individual species, such as *C. acnes*, but rather arises from complex and dynamic microbial interactions within the skin microbiome [69]. The synergistic virulence between *C. acnes* and *Staphylococcus*, coupled with the opportunistic proliferation of *Streptococcus* under antibiotic pressure, highlights the importance of therapeutic approaches aimed at restoring microbial balance rather than simply targeting dominant bacterial populations.

The CAMP factor family comprises secreted proteins that induce the CAMP reaction, a haemolytic process potentially linked to bacterial virulence [48]. Despite low sequence homology between CAMP factors from *C. acnes* and other bacteria, these proteins are expressed at significantly higher levels in acne lesions compared with non-acne skin [70,71]. Our functional comparison of four putative CAMP factors revealed distinct haemolytic properties, adding a new dimension to our comprehension of the molecular mechanisms driving acne. *In vivo* experiments further confirmed that CAMP3 and CAMP5 are pivotal in promoting acne-like inflammation and sebaceous gland atrophy, both of which are implicated in the formation of comedones and acne inversa [72,73]. Their haemolytic activity and ability to induce pro-inflammatory cytokines, such as IL-6 and IL-8, suggest a complex role in acne pathogenesis [74]. Targeting specific CAMP factors and their associated inflammatory pathways may offer a promising strategy for acne treatment and prevention of recurrence.

In animal models, we observed that CAMP stimulation upregulates IL-8 expression in a dose-dependent manner, while topical inhibition of IL-8 *in vivo* led to a concurrent reduction in CAMP levels. This bidirectional association raises the possibility of a positive feedback loop between CAMP factors and IL-8 signalling. Supporting this, previous research has demonstrated that monoclonal antibodies against the *C. acnes* CAMP factors significantly reduced IL-8 production in inflamed acne tissues, further validating CAMP as a driver of cytokine-mediated inflammation [71]. These findings collectively suggest that CAMP-induced cytokine release, particularly IL-8, may in turn sustain or amplify CAMP expression under inflammatory conditions. However, it is also possible that the observed reduction in CAMP and IL-8 following anti-inflammatory treatment reflects the downstream resolution of inflammation rather than a direct regulatory loop [75]. Further mechanistic studies are warranted to clarify this relationship. Nonetheless, the interplay between CAMP and IL-8 highlights their potential as synergistic therapeutic targets for modulating acne-related inflammation.

Interestingly, our cellular assays demonstrated that merely CAMP3 and CAMP5 significantly induced IL-8 expression, in contrast to the broader response observed *in vitro*. This discrepancy may reflect differences in local immune context, protein stability or tissue penetration *in vivo* and underscores the importance of validating *in vitro* findings in physiologically relevant models [76]. It also suggests that among the CAMP family, only specific members may be functionally relevant in driving IL-8-mediated inflammation in acne lesions [54].

Finally, we determined the crystal structure of the *C. acnes* CAMP3 factor at a resolution of 2.43 Å. This high-resolution structure reveals a characteristic CAMP-like fold, emphasizing the conserved architectural features shared across this protein family. However, significant questions remain regarding the structural and functional diversity among other CAMP proteins in the * C. acnes* family. Comparative structural studies and functional assays will be crucial in elucidating these differences, potentially uncovering novel insights into their roles in microbial physiology and pathogenicity.

## Conclusions

In summary, our study reveals significant microbial dysbiosis in early acne lesions, underscoring a marked imbalance within the microecology of comedones. We compared the virulence of various CAMP factors from *C. acnes*, assessing both their pro-inflammatory and sebaceous gland differentiation effects *in vitro* and *in vivo*. Additionally, structural analysis of CAMP3 provided deeper insight into its role in acne pathogenesis. Together, these findings clarify the functional diversity of *C. acnes* CAMP factors and highlight potential new avenues for targeted acne therapy.

While this study provides valuable insights into the role of *C. acnes* and its CAMP factors in acne pathogenesis, several limitations should be acknowledged. First, although the structural analysis of CAMP factors may offer limited novelty, given the previously resolved structure of a closely related CAMP factor from *M. curtisii* and *S. agalactiae* [21,25], our study provides a comparative analysis across different *C. acnes* strains. This comparison underscores the potential functional diversity of CAMP factors, which may contribute to their varying pathogenic roles in acne development. Second, while our findings indicate that IL-8-neutralizing antibody therapy effectively reduces inflammation in a murine model, its clinical efficacy, mechanisms and feasibility for topical application in humans remain unclear. Further investigations are needed to confirm its therapeutic potential, optimize delivery systems and evaluate its long-term effects on human skin. Third, our study primarily focuses on CAMP factors and their impact on keratinocytes and sebaceous glands. A more comprehensive understanding of acne pathogenesis will require a broader exploration of other virulence factors and host–microbe interactions. Additionally, variability in CAMP expression across different * C. acnes* strains and its clinical relevance in diverse patient populations warrant further investigation. Another limitation is that we utilized microbial strains obtained from ATCC, which may differ genetically from strains found in actual human skin microbiota. In future studies, we plan to examine the impact of *C. acnes* strains collected from human skin.

By addressing these limitations, future research can build upon our findings to advance the understanding of acne pathogenesis and contribute to the development of more effective therapeutic strategies.

## Supplementary material

10.1099/mgen.0.001449 Uncited Table S1.

10.1099/mgen.0.001449 Uncited Table S2.

10.1099/mgen.0.001449 Uncited Table S3.

10.1099/mgen.0.001449 Uncited Table S4.

10.1099/mgen.0.001449 Uncited Supplementary Material 1.

## Abbreviations

ANOSIM: analysis of similarities
*C. acnes*: *Cutibacterium acnes*
CAMP: Christie–Atkins–Munch-Peterson
ICR: Institute of Cancer Research
KEGG: Kyoto Encyclopedia of Genes and Genomes
LDA: linear discriminant analysis
LEfSe: linear discriminant analysis effect size
PCoA: principal coordinate analysis
RMSD: root-mean-square deviation
RT-qPCR: quantitative real-time PCR
SCFAs: short-chain fatty acids
SMD: standard mean difference
SRBC: sheep red blood cell
