# Supplementary material for: Insights into microbial dysbiosis and Cutibacterium acnes CAMP factor interactions in acne vulgaris
Source: Microb Genom. 2025 Jul 16;11(7):001449. doi: 10.1099/mgen.0.001449 (PMC12265948; doi:10.1099/mgen.0.001449)
Supplement: Uncited Supplementary Material 1. [file mgen-11-01449-s005.pdf]

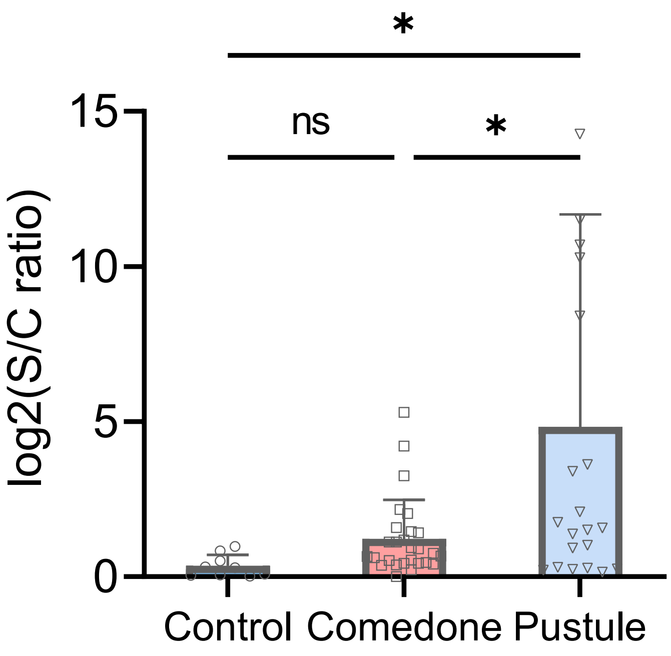

FigS1. Increased *Staphylococcus*/*C. acnes* (S/C) ratio in acne lesions
The *Staphylococcus*/*C. acnes* (S/C) ratio is calculated and compared among groups to suggest a potential link to an inflammatory state, as determined by 16S rRNA gene sequencing (*$P < 0.05$, ns = not significant).

a Acne samples

Study	Total	ZCOR	95%−CI	Weight (common)	Weight (random)
Schneider AM 2023	27	0.67	[0.27; 1.07]	6.7%	7.4%
Nolan ZT 2023	15	1.15	[0.58; 1.72]	3.4%	6.0%
Zhou L 2022	74	0.12	[−0.11; 0.35]	19.9%	8.9%
Hrapovic N 2022	18	0.93	[0.42; 1.43]	4.2%	6.5%
Cavallo I 2022	10	0.47	[−0.27; 1.21]	2.0%	4.7%
Xu XX 2021	14	0.26	[−0.33; 0.85]	3.1%	5.8%
Thompson KG 2021	8	0.19	[−0.68; 1.07]	1.4%	3.8%
Loss M 2021	14	0.70	[0.10; 1.29]	3.1%	5.8%
Liu JX 2021	20	0.05	[−0.42; 0.53]	4.8%	6.8%
Huang RY 2021	15	0.14	[−0.43; 0.70]	3.4%	6.0%
Li CX 2019	67	0.42	[0.17; 0.66]	18.0%	8.8%
Dagnelie MA 2019	24	0.51	[0.09; 0.94]	5.9%	7.2%
Dreno B 2017	26	0.09	[−0.31; 0.50]	6.5%	7.4%
Park SY 2020	20	0.20	[−0.27; 0.68]	4.8%	6.8%
Fitz−Gibbon S. 2013	49	1.33	[1.04; 1.62]	12.9%	8.4%
**Common effect model**	**401**	**0.49**	**[0.39; 0.59]**	**100.0%**	**--**
**Random effects model**		**0.49**	**[0.27; 0.71]**	**--**	**100.0%**

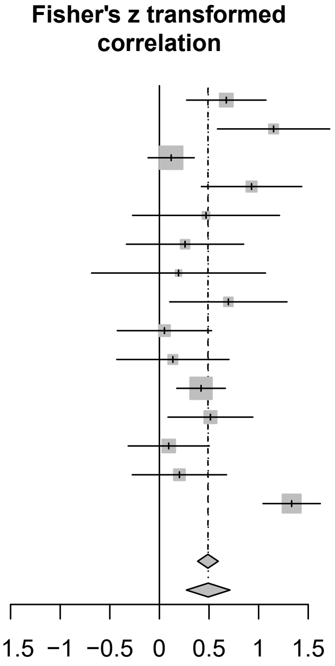

Heterogeneity: $I^2 = 78\%$, $\tau^2 = 0.1278$, $p < 0.01$

b Control samples

Study	Total	ZCOR	95%−CI	Weight
Schneider AM 2023	21	0.22	[−0.24; 0.68]	19.0%
Zhou L 2022	19	0.08	[−0.41; 0.57]	17.9%
Thompson KG 2021	8	0.29	[−0.59; 1.17]	8.4%
Loss M 2021	14	0.85	[0.26; 1.44]	14.5%
Liu JX 2021	19	0.06	[−0.43; 0.55]	17.9%
Li CX 2019	9	0.51	[−0.29; 1.31]	9.6%
Dagnelie MA 2019	12	1.00	[0.35; 1.65]	12.8%
**Random effects model**	**102**	**0.39**	**[0.10; 0.68]**	**100.0%**

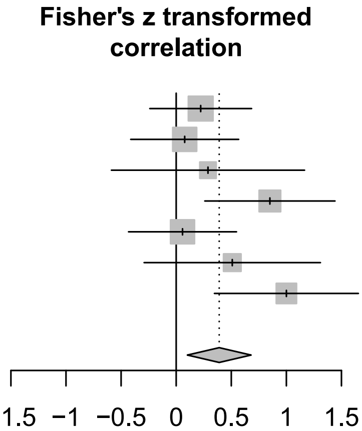

Heterogeneity: $I^2 = 37\%$, $\tau^2 = 0.0591$, $p = 0.14$

c Acne vs. control samples

Study	Experimental Total	Experimental Mean	Experimental SD	Control Total	Control Mean	Control SD	SMD	95%−CI	Weight
Schneider AM 2023	27	0.59	0.3899	21	0.22	0.3341	0.99	[0.38; 1.60]	17.9%
Zhou L 2022	74	0.12	0.1247	19	0.08	0.1266	0.32	[−0.18; 0.83]	19.2%
Thompson KG 2021	8	0.19	0.1546	8	0.28	0.1856	−0.50	[−1.50; 0.50]	12.9%
Loss M 2021	14	0.60	0.3848	14	0.69	0.4299	−0.21	[−0.96; 0.53]	16.1%
Liu JX 2021	20	0.05	0.0490	19	0.06	0.0590	−0.11	[−0.74; 0.52]	17.6%
Dagnelie MA 2019	24	0.47	0.3542	12	0.76	0.1614	−0.92	[−1.65; −0.19]	16.3%
**Random effects model**	**167**			**93**			**−0.03**	**[−0.57; 0.51]**	**100.0%**

Heterogeneity: $I^2 = 74\%$, $\tau^2 = 0.3333$, $p < 0.01$

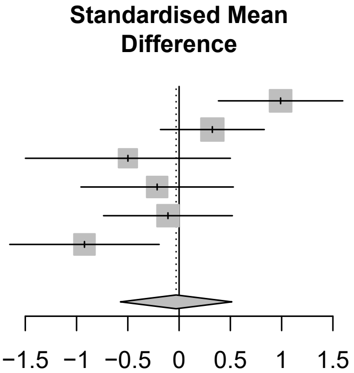

FigS2. Meta analysis shows no differences of *C. acnes* abundance between acne and control groups

a-b. Forest plot of *C. acnes* abundance in acne and control groups.

c. Comparison of *C. acnes* abundance between acne and control groups. The squares indicate individual studies. The diamonds represent pooled effect sizes. The dashed lines represent the 95% CIs. Data were analyzed separately by using a random-effects model.

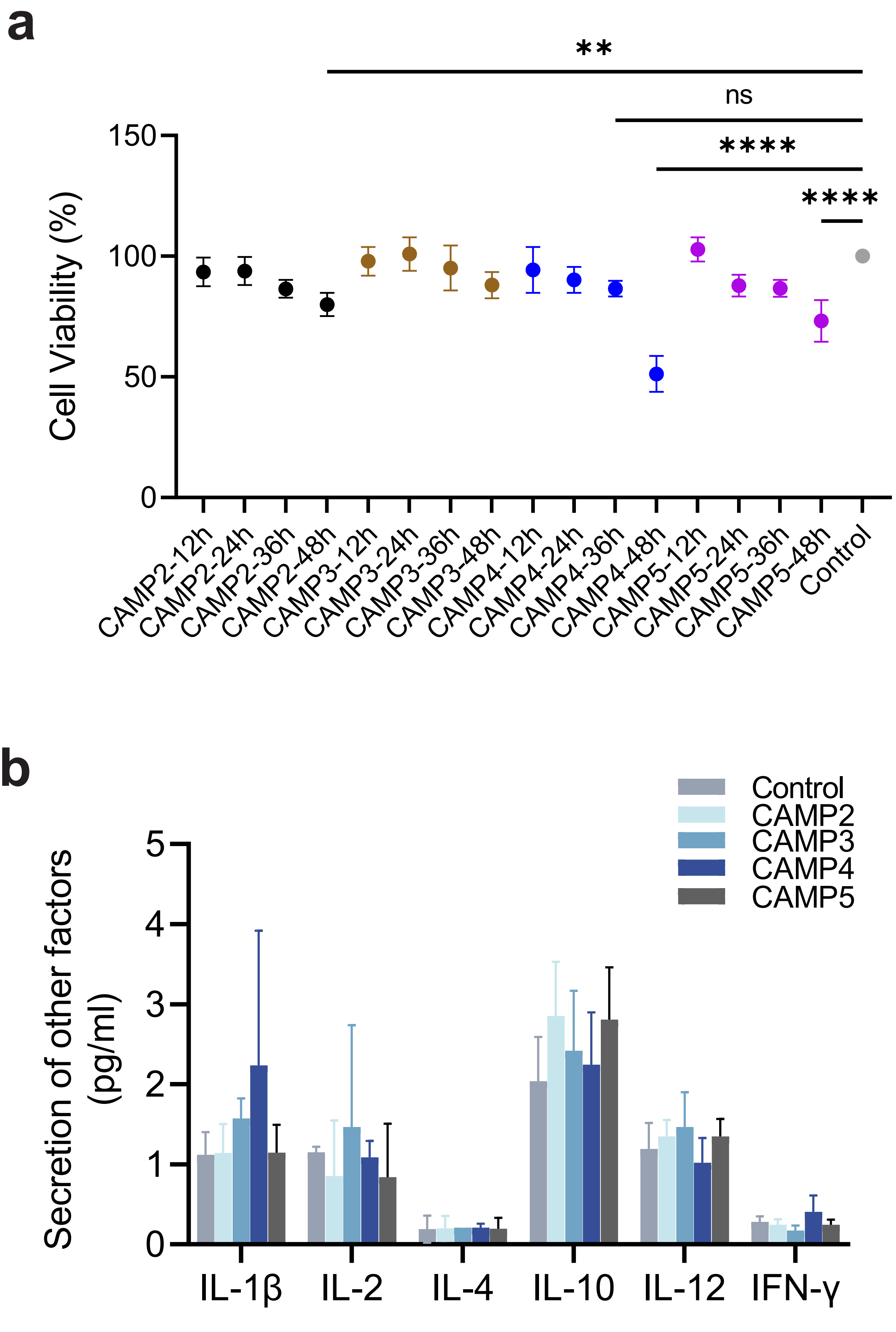

FigS3. Cell viability and cytokine induction in keratinocytes following stimulation with CAMP proteins.

a. Time-course analysis of cell viability following CAMP protein stimulation in keratinocytes. Keratinocytes were treated with CAMP proteins for 12, 24, 36, or 48 hours, and cell viability was evaluated using the CCK-8 assay. Data are shown as mean ± SEM (n = 3, ****P < 0.0001, **P < 0.01, ns = not significant).

b. Cytokine levels in response to CAMP proteins stimulation (10 μM). Control is shown for comparison.

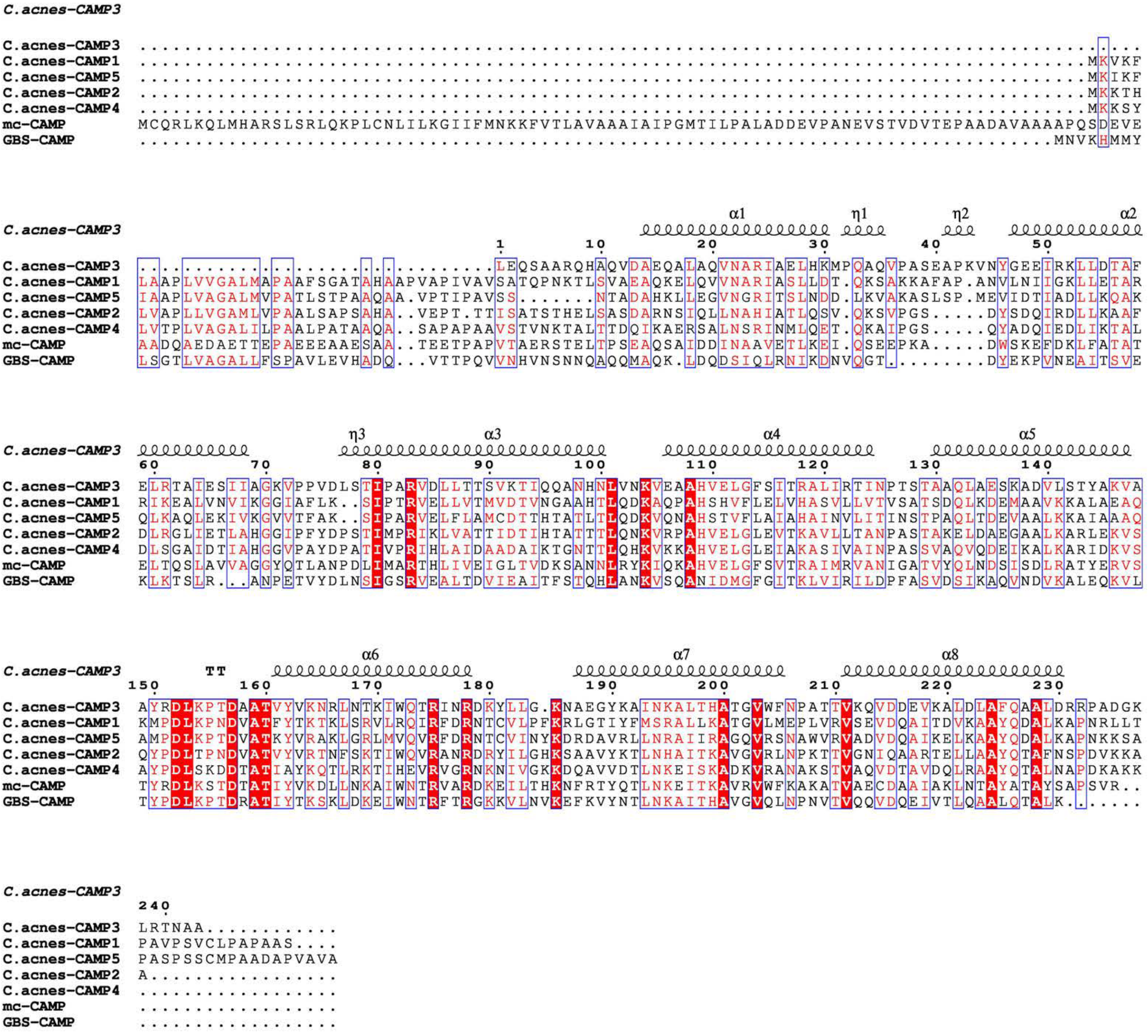

FigS4. Sequence alignment of CAMP factors from different bacteria

Secondary structure elements of *C.acnes* CAMP3 are indicated above the sequence. Strictly conserved residues are colored dark red, while conservative substitutions are depicted with box.
